# A feasibility study of a decision aid to support family carers of people with severe dementia or those towards the end-of-life

**DOI:** 10.1177/02692163221122379

**Published:** 2022-09-08

**Authors:** Nathan Davies, Narin Aker, Victoria Vickerstaff, Elizabeth L Sampson, Greta Rait

**Affiliations:** 1 Centre for Ageing Population Studies, Research Department of Primary Care and Population Health, University College London, London, UK; 2 PRIMENT Clinical Trials Unit, Research Department of Primary Care and Population Health, University College London, London, UK; 3 Marie Curie Palliative Care Research Department, Division of Psychiatry, University College London, London, UK; 4 Department of Psychological Medicine, Royal London Hospital, East London NHS Foundation Trust, London, UK

**Keywords:** Dementia, caregivers, decision support techniques, palliative care, end of life care

## Abstract

**Background:**

Advance care planning in dementia does not always happen. As dementia progresses, decisions are often left for family carers to make with professionals.

**Aim:**

To test the feasibility and acceptability of the delivery and use of a decision aid for family carers of people with severe dementia or towards the end-of-life.

**Design:**

Feasibility study using a before-after design of a paper-based decision aid with family carers of people with severe dementia or towards the end-of-life. Criteria for whether to progress to full evaluation included achieving: 70% recruitment rate of target of 30 people, and retention of 70% at 6 months. Outcome measures at baseline, 3 and 6 months, included: the Decisional Conflict Scale (DCS), Kessler Psychological Distress Scale (K10), EQ5D-5L and Satisfaction with Care at the End of Life (SWC-EOLD).

**Participants:**

Twenty-eight family carers were recruited (93% of target), 26 completed baseline assessment and 20 (71%) of those were followed-up at 6 months.

**Results:**

Almost all outcomes changed indicating improvement over 6 months. The DCS and K10 scores decreased indicating less decisional conflict and less psychological distress. The decision aid was acceptable, 25% found it very helpful and 55% a little helpful at 6 months.

**Conclusion:**

We met the success criteria demonstrating this study was feasible and acceptable to carers. Future research should test the effectiveness of the decision aid in a full scale evaluation.

**What is already known about this topic?**

- Advance care planning does not always happen with people living with dementia and as dementia progresses they are less able to participate in decision making.
- Family carers find making decisions emotional and difficult, especially decisions about end-of-life care.
- There are a lack of decision aids which focus on more than one decision in dementia care.

**What this paper adds?**

- A decision aid with multiple decisions in dementia care is acceptable to family carers.
- It is feasible to test a decision aid for family carers of people living with severe dementia or towards the end-of-life, including being able to recruit and retain participants over 6 months.

**Implications for practice, theory or policy**

- A full-scale evaluation of this decision aid is warranted to evaluate effectiveness.

## Introduction

Despite efforts to increase advance care planning for people with dementia,^1^ this does not always happen.^2^ As dementia progresses, people with dementia are less likely and able to be involved in making decisions.^3^ In the UK the Mental Capacity Act states decisions about those who lack capacity must be made in the person’s best interest.^4^ Decisions may include decisions about everyday wellbeing, moving to a care home, or even some end-of-life decisions with professionals about starting or stopping medical treatments.^5,6^ Carers often feel responsible for making decisions,^5,7,8^ and find decision making difficult and emotional,^9^ especially decisions about end-of-life care.^5^

Family carers may benefit from support in making decisions.^8,10,11^ Decision aids are effective to support decision making among patients and family carers including improving patient knowledge and expectations.^12,13^ Decision aids explicitly state the decision, provide information about the decision and summarise options along with associated benefits and harms.^12^

Existing dementia care decision aids either focus on single decisions such as place of care, or topics such as goals of care which can encompass several broad decisions about care.^14,15^ When caring for someone with dementia towards end-of-life, family carers are often faced with multiple, interrelated decisions.^14^ There is a need for a decision aid which covers this variety of decisions and topics including, specifically, place of care, as well as broader decisions about care approaches, considering the complexities faced by carers towards the end of life. We developed a decision aid to support family carers making decisions on behalf of the person with severe dementia or those towards the end-of-life, covering multiple decisions.^16^

## Aims

To test the feasibility and acceptability of delivering and use of a decision aid for family carers of people with severe dementia or those towards the end-of-life.

## Methods

### Design

Six-month feasibility study using a before-after design with a target of 30 family carers, reported using the CONSORT 2010 statement extension for pilot and feasibility studies.^17^

### The decision aid intervention

A paper-based decision aid for family carers providing care for someone with severe dementia or those towards the end-of-life living at home or in a nursing home. The decision aid includes four key decision topics: (1) changes in care; (2) eating and drinking difficulties; (3) everyday well-being and (4) healthcare, tests and medication. Carers can record their preference for these decisions. Detailed information on the co-design of the decision aid and content are published separately.^16^

### Sample and recruitment

Family carers of people with severe dementia or towards the end-of-life were recruited between April 2020 and October 2020 through:

(1) Local and national dementia, carer, palliative care, research and professional networks;
(2) NIHR Join Dementia Research (JDR): an online dementia research registry;
(3) Social media.

Interested potential participants were asked to respond using any means of communication to either the research team or the inviting organisation.

Our protocol also included recruitment via NHS hospital trusts and nursing homes. However, the study commenced in April 2020 and due to Covid-19 we did not recruit via these sites.

### Inclusion criteria

Family carers:

- Family member or friend over the age of 18 years who identified as a carer/main decision maker for someone with severe dementia or towards the end-of-life who lives at home in the community or in a nursing home;
- Able to provide informed consent;
- Able to read and speak English.

The person with dementia being cared for had to:

- Be over the age of 65 years;
- Have a clinical diagnosis of any type of dementia as categorised in ICD-11 (informed by clinical record or family carer);
- Judged by the research or clinical team to be towards the end-of-life, have severe dementia or live in a nursing home.

End-of-life is difficult to predict and define in dementia,^18^ we therefore devised a broad overview of the characteristics of someone with dementia who may be towards end-of-life. These were pragmatically informed by the Clinical Dementia Rating (CDR) score of 3 for severe dementia.^19^ Not everyone who is towards the end-of-life may have severe dementia and may not experience the symptoms listed below. A clinical judgement was made by clinical team members about the stage of dementia and not by formal rating. Someone with dementia towards the end-of-life is likely be to be physically frail and experience repeated instances of illness. We considered a combination of two or more of these as an indication that someone with dementia may be near the end-of-life:

- Severe memory loss;
- Unable to make judgements or solve problems including an inability to communicate;
- Increasing frailty and reduced mobility, becoming bed bound;
- Recurrent episodes of infections (i.e. chest infections);
- Recurrence of bed sores;
- Eating less, swallowing difficulties and loss of weight;
- Bedbound;
- Frequent incontinence;
- Requires much help with personal care;
- Other severe or life limiting illness (i.e. cancer or lung disease).^19,20^

### Exclusion criteria

- Family carers who had a cognitive impairment themselves;
- Family carers not living in the UK

### Sample size calculation

No formal sample size calculation was undertaken, as this is a feasibility study. Numbers were chosen on pragmatic grounds to demonstrate feasibility of recruitment, acceptance of the intervention and retention. We aimed to recruit 30 carers, and assuming 14% attrition anticipated that 26 participants would complete the study. About 14% attrition is based on similar studies of people with dementia towards the end-of-life.^21,22^

### Ethical approval and consent

London – Queen Square Research Ethics Committee and Heath Research Authority approved the study March 2020 (20/LO/0210). We collected written or recorded verbal consent.

### Measures

A research assistant completed baseline assessments and follow-up assessments at 3 and 6 months via telephone. Measures included:

- Decisional Conflict Scale (DCS): to evaluate the quality of a decision about care.^23^
- Kessler Psychological Distress Scale 10 (K-10): providing a global measure of distress.^24^
- Satisfaction with Care at the End of Life (SWC-EOLD).^25^
- Carers Quality of Life measured using EQ-5D-5L, EuroQol.^26^

We used the DCS twice per assessment: (1) participants were asked to answer the question reflecting on one decision (single decision) included in the decision aid they had made and (2) participants were asked to reflect on all decisions they were currently making (all decisions).

We collected data on the acceptability of being involved in the study including assessment length, helpfulness of the study and any distress caused.

### Analysis

Data analysis was descriptive. Summary measures are presented for the baseline characteristics as mean and standard deviations for continuous variables and frequencies and percentages for categorial variables. The summary results are based on observed observations only. We estimated changes in pre- and post-intervention scores (with confidence intervals). We pre-defined success criteria for determining whether it is appropriate to progress to a full evaluation (see Table 1).

**Table 1.** Success criteria for full evaluation.

Success criteria	Achieved
A recruitment rate of a minimum 70% of our target (30 participants)	28 consented. 93% of target recruitment rate.
A retention rate of 70% at 6 months	20/28 at 6 months. 71% retention rate.
A positive evaluation of feasibility and acceptability to family carers	Questionnaires were completed with no missing items on any of the questionnaires and positive evaluation (see Table 3)

## Results

### Sample

**Figure 1.**
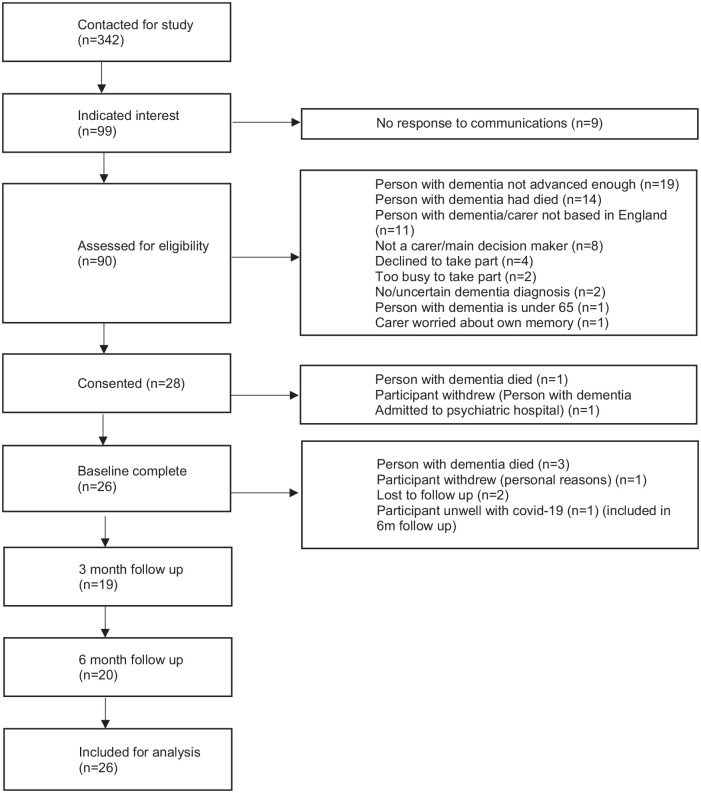
Flow chart of study participants.

Twenty-eight participants (see Table 2) from a target of 30 (93% of target) consented to be part of the study. Figure 1 shows flow of participants which includes reasons for withdrawal.

**Table 2.** Participant demographics.

Demographic information, *N* = 26	PWD, *N* = 26	Carer, *N* = 26
Results are displayed as *n* (%), unless specified otherwise		
Age, mean (SD)	82.8 (9.4)	61.2 (12.8)
Gender, female	15 (58)	24 (92)
Ethnicity		
White	24 (92)	25 (96)
Other	2 (8)	1 (4)
Marital status		
Married	10 (38)	15 (58)
Divorced	3 (12)	2 (8)
Cohabiting	1 (4)	2 (8)
Widowed	12 (46)	1 (4)
Single	0 (0)	6 (23)
Sexuality		
Heterosexual or straight	26 (100)	25 (96)
Don’t know or prefer not to say		1 (4)
Residence		
Owner-occupied	–	25 (96)
Housing association rented	–	1 (4)
Private home, no health services	9 (35)	–
Private home, with social services	10 (38)	–
Residential home	4 (15)	–
Other nursing home	2 (8)	–
Other (sheltered accommodation)	1 (4)	–
First language		
English	24 (92)	26 (100)
Other	2 (8)	0 (0)
Highest level of education		
No qualifications	10 (38)	–
O levels	2 (8)	1 (4)
A levels (or post O level)	6 (23)	2 (8)
Degree	4 (15)	14 (54)
Postgraduate	4 (15)	9 (35)
Religion		
Christian	19 (73)	10 (38)
Other	3 (12)	2 (8)
No specific	4 (15)	14 (54)
Lives with		
Alone	5 (19)	
Spouse	7 (27)	
Child	3 (12)	
Other	5 (19)	
N/A	6 (23)	
Relationship to person with dementia		
Spouse		8 (31)
Child		14 (54)
Sibling		1 (4)
Other		3 (12)
Dementia type		
Alzheimer’s disease	13 (50)	
Frontotemporal dementia	3 (12)	
Vascular dementia	5 (19)	
Dementia with Lewy bodies	2 (8)	
Mixed dementias	1 (4)	
Other	2 (8)	
Time since dementia diagnosis, years	5.8 (3.8)	
Psychiatric history		
Depression (inc. post-natal depression)	9 (35)	
None	17 (65)	

## Acceptability

No participants felt the study caused ‘significant distress’. Only two participants at baseline and 1 at 3 months found it a ‘little distressing’. None were distressed at 6 months (See Table 3). At 6 months 25% reported they found it very helpful and 55% a little helpful. Detailed acceptability information was collected from semi-structured qualitative interviews and is reported separately.

**Table 3.** Participant experience and acceptability.

	Baseline, *N* = 26	3 months, *N* = 19	6 months, *N* = 20
	*n* (%)	*n* (%)	*n* (%)
Do you feel you could cope with the length of the assessment?			
Yes, quite easily	19 (73)	16 (84)	16 (80)
Only just	7 (27)	3 (16)	4 (20)
No	0 (0)	0 (0)	0 (0)
Did you find taking part in the study helpful?			
Yes, very	5 (19)	9 (47)	5 (25)
A little helpful	17 (65)	6 (32)	11 (55)
No	4 (15)	4 (21)	4 (20)
Did you feel the study caused you distress?			
Yes, a lot	0 (0)	0 (0)	0 (0)
A little	2 (8)	1 (5)	0 (0)
No	24 (92)	18 (95)	20 (100)

## Outcomes measures

Scores across all outcome measures can be seen in Table 4, improvements were recorded in decisional conflict, distress and satisfaction with care, but not in quality of life. See Box 1 for the decision topics participants selected and the preference they reported for these decisions when answering the DCS for a single decision.

**Table 4.** Outcomes measures.

	Descriptive						Pre-post analysis, *n* = 20 (6 months–baseline)	
	*n*	Baseline mean (SD)	*n*	3 months, mean (SD)	*n*	6 months, mean (SD)	Mean diff.	95% CI
DCS (single decision)								
Total	26	33.7 (13.1)	19	25.1 (15.3)	20	27.7 (14.6)	−2.34	(−9.46, 4.77)
Sub-scores								
Uncertainty	26	40.1 (22)	19	29.8 (19.5)	20	32.1 (19.7)	−4.17	(−15.4, 7.11)
Informed	26	34.9 (20.8)	19	24.1 (18.2)	20	26.7 (17.6)	−1.25	(−10.4, 7.90)
Values clarity	26	27.2 (13.7)	19	15.8 (13.3)	20	22.1 (15.8)	−4.58	(−14.7, 5.58)
Support	26	42.9 (23.8)	19	38.6 (23.8)	20	35.8 (19.1)	−3.33	(−14.0, 7.36)
Effective decision	26	26 (14.7)	19	19.1 (16.2)	20	23.4 (15.8)	0.63	(−8.27, 9.52)
DCS (all)								
Total	26	32.3 (11.3)	19	26 (14.6)	20	26.6 (14)	−4.14	(−8.80, 0.52)
Sub-scores								
Uncertainty	26	42 (19.2)	19	37.3 (20.7)	20	35 (19.6)	−6.67	(−15.2, 1.88)
Informed	26	30.8 (17.4)	19	21.5 (15.5)	20	23.8 (15.6)	−2.50	(−9.33, 4.33)
Values clarity	26	25.3 (12.6)	19	20.2 (14.8)	20	19.2 (12.1)	−5.00	(−11.5, 1.50)
Support	26	38.8 (19.7)	19	30.3 (24.1)	20	35.4 (19.3)	−3.33	(−9.58, 2.92)
Effective decision	26	26.4 (10.9)	19	22 (14.2)	20	21.3 (14.1)	−3.44	(−9.55, 2.67)
Kessler-10	26	18.9 (7.2)	19	16.6 (6.8)	20	17.6 (6.3)	−0.80	(−3.13, 1.52)
SWC-EOLD	26	27.8 (3.9)	19	27.4 (3.7)	20	28.5 (2.6)	0.30	(−1.85, 2.45)
EQ-5D	26	0.9 (0.1)	19	0.9 (0.1)	20	0.9 (0.2)	−0.04	(−0.09, 0.011)

**Box 1.** Decisions and preferences made by carers.

*Decision topic chosen when answering the DCS for single decision*
‘Changes in care’ (*n* = 16)
‘Everyday wellbeing of the person dementia’ (*n* = 5)
‘Healthcare tests and medication’ (*n* = 3)
‘Eating and drinking’ (*n* = 2)
*Preferences made by carers*
‘Using power of attorney’ (*n* = 1), ‘avoid hospital admission’ (*n* = 1), ‘accept hospital admission but with no ventilator’ (*n* = 1), ‘move to care home’ (*n* = 6), ‘arrange respite’ (*n* = 1), ‘no more solid food’ (*n* = 1), ‘feed by hand’ (*n* = 1), ‘quality over quantity for food’ (*n* = 1), ‘cancel health insurance’ (*n* = 1) ‘sell individual’s house’ (*n* = 1), ‘provide personal care closer to bedroom (mobility issues)’ (*n* = 1), ‘not to visit during COVID-19 lockdown’ (*n* = 1), ‘DNAR’ (*n* = 1), ‘not to involve or stop care agency’ (*n*=1), ‘not to move into a care home’ (*n*=1), ‘care at home’ (*n* = 1), ‘live in carer’ (*n* = 1), ‘paid carers at home’ (*n* = 1), ‘keep same carer’ (*n* = 1), ‘live in carer’ (*n*=1), ‘stop carer agency’ (*n* = 1)

DCS (Decisional Conflict Scale) range 0–100, higher score indicates high decisional conflict; Kessler 10: range 10–50, higher scores indicate a higher level of psychological distress; SWC-EOLD (satisfaction with care end-of-life dementia), range 10–40, higher scores indicating more satisfaction; EQ-5D, range 0–1, higher scores indicate better health.

## Discussion

### Main findings

We demonstrated our intervention is feasible and acceptable to family carers supporting someone with severe dementia or towards end-of-life. This was a feasibility study so not powered to report effectiveness, but results indicated that decisional conflict reduced post intervention for both individual decisions (standardised effect size 0.6) and when asked to consider all the decisions they had made (standardised effect size 1.7). These are larger than the meaningful difference of 0.3–0.4. recommended in the DCS manual. This suggests the decision aid supported participants’ confidence and certainty about making decisions. Measures of distress and satisfaction with care also improved.

Our results support previous studies which have demonstrated similar effects when using decision aids to support family carers of people living with dementia make specific decisions,^14^ but also suggest decision aids are able to cover more than one topic.

Many potential participants were keen to participate but did not meet the eligibility criteria of end-of-life or severe dementia. Future evaluation could widen the inclusion criteria to include all stages of dementia where carers feel they are contributing to decision making.

*What this study adds* Meeting the success criteria supports a larger fully powered evaluation of the decision aid using the outcomes from this study. The decision aid has the potential to support planning for palliative care but also real-time decisions about palliative care. The DCS may not be best suited for this decision aid covering multiple topics and decisions. The DCS is designed to focus on single decisions, however participants in this study were considering multiple decisions with multiple potential options/preferences. We therefore measured the DCS both on a single decision participants made (based on their choice, see Box 1), as well as separately to record their experiences of general decision making. Future work should explore the use of the DCS or similar measure to cover multiple decisions.

### Strengths and limitations

Most participants were white, a larger evaluation study would need to increase sample diversity and ensure underserved populations are included. Using online recruitment methods meant we were able to broaden the geographical scope, ensuring we were not limited to participants in highly researched areas such as London, but may still have excluded some people. A detailed qualitative evaluation will be published separately. Convenience sampling due to the covid pandemic meant most participants had already made some of the decisions in the decision aid and used the aid to reflect on decisions and plan for future decisions. If we had recruited via the NHS or care homes, we may have identified carers currently making decisions in the decision aid.

## Conclusion

Our decision aid was feasible and acceptable to family carers. We were able to successfully recruit and follow up family carers over 6 months. A larger evaluation is warranted to test effectiveness of the decision aid.
